# The Complex Quest of Preventing Obesity in Early Childhood: Describing Challenges and Solutions Through Collaboration and Innovation

**DOI:** 10.3389/fendo.2021.803545

**Published:** 2022-02-07

**Authors:** Anna Lene Seidler, Brittany J. Johnson, Rebecca K. Golley, Kylie E. Hunter

**Affiliations:** ^1^ National Health and Medical Research Council Clinical Trials Centre, University of Sydney, Camperdown, NSW, Australia; ^2^ Transforming Obesity Prevention in CHildren (TOPCHILD) Collaboration, Sydney, NSW, Australia; ^3^ Caring Futures Institute, Flinders University, Bedford Park, SA, Australia; ^4^ College of Nursing and Health Sciences, Flinders University, Bedford Park, SA, Australia

**Keywords:** children, obesity prevention, family, intervention, innovative methods

## Abstract

Childhood obesity remains a major public health issue and priority area for action. Promisingly, obesity prevention interventions in the first 2000 days of life have shown modest effectiveness in improving health behaviours and healthy weight status in children. Yet, researchers in this field face several challenges. This can lead to research waste and impede progress towards delivering effective, scalable solutions. In this perspective article, we describe some of the key challenges in early childhood obesity prevention and outline innovative and collaborative solutions to overcome these. Combining these solutions will accelerate the generation of high-quality evidence that can be implemented into policy and practice.

## Introduction

Globally, the prevalence of overweight and obesity in early childhood has continued to increase from an estimated 30.3 million (4.9%) children aged under 5 years in 2000 up to 38.3 million (5.6%) in 2019 (1). Early obesity can set children on a lifelong negative health trajectory, since children with obesity are more likely to have obesity as adults and be afflicted by associated health conditions (2, 3). There are numerous known and unknown factors influencing obesity risk ranging from genetic and epigenetic to behavioural, social and environmental factors (4). Health behaviours are one major influence on obesity risk, and unlike other factors (e.g. genetic factors) health behaviours can be modified by interventions (5). The first 2000 days are an important life stage for the prevention of obesity when children learn a range of behaviours relating to diet, movement and sleep (6, 7). Parents and caregivers are a major influence at this stage, and their health behaviours influence their children’s health behaviours (8–10). Therefore parent-focused behavioural interventions are a key strategy for early childhood obesity prevention (11), and interventions often involve a family approach that includes parents improving their own health behaviours.

Previous reviews have demonstrated that parent-focused behavioural early obesity prevention interventions can be effective (5, 12–14). A Cochrane systematic review found moderate-certainty evidence that combined dietary and physical activity interventions can lead to a small reduction in body mass index (BMI) in children aged 0-5 years (mean difference -0.07, 95% CI -0.14 to -0.01) (5). The Early Prevention of Obesity in Childhood (EPOCH) Prospective Meta-Analysis combined row-by-row individual participant data from four Australasian randomised controlled trials with a total of 2196 mother-infant dyads (15). They found that compared to usual care, behavioural interventions starting early (in pregnancy or the first 6 months after birth) were effective in reducing relative weight (BMI z-score) at 1.5-2 years by 0.12 standard deviations (95% CI, -0.22 to -0.02). On a population level, this would equate to a decrease in obesity prevalence of about 2%. Overall, these past reviews point to the potential of early interventions in reducing overweight and obesity, but also opportunities to enhance their effectiveness, since effect sizes were often small.

With the importance of early intervention apparent, a burgeoning number of early obesity prevention trials have commenced in recent years. Worldwide, we have identified more than 70 obesity prevention trials commencing during pregnancy or within the first year after birth, with a total combined sample size of about 55,000 participants (further details of this search and the studies identified are available elsewhere) (16). Yet, despite extensive research efforts uncovering promising interventions to reduce obesity risk, global obesity rates are still on the rise. This perspective piece aims to describe key challenges in early childhood obesity prevention research and propose innovative solutions to move forward in the quest to address this major public health issue.

## Challenges

**Figure 1:**
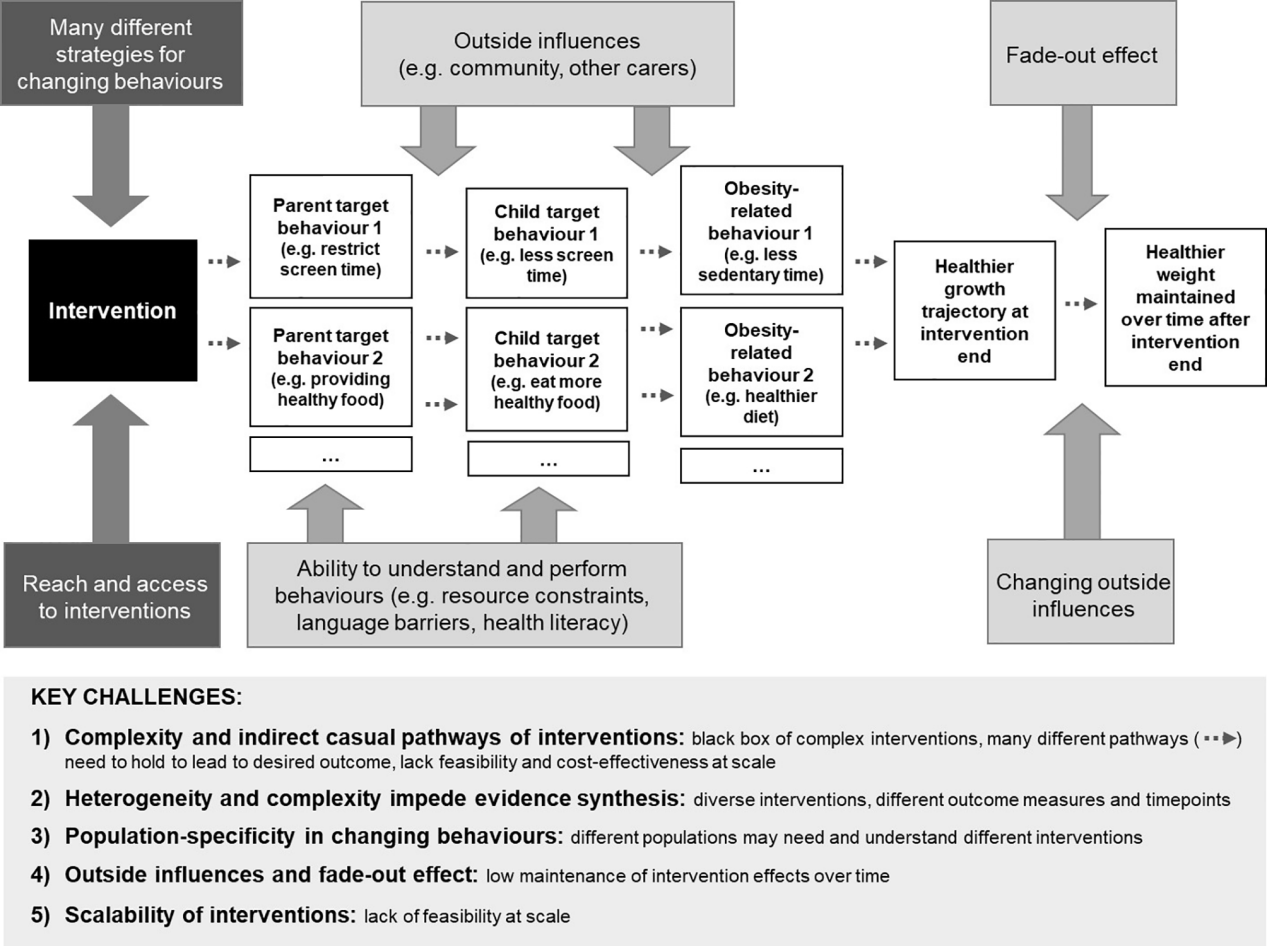
Simplified illustration of intervention pathways and challenges for effective childhood obesity prevention. The ‘Key challenges’ box at the bottom of the figure summarises the challenges arising from the complex pathway depicted in the top section of the figure.

**Figure 1** provides an overview of the key challenges in early childhood obesity prevention.

### Challenge 1: Complex and Indirect Causal Pathways of Interventions

Promising interventions are often complex, and target multiple behaviours using a diverse range of behaviour change strategies (17). In addition, the dose of behaviour change content received and implemented by participants remains unclear. Consequently, it is difficult to disentangle the components of interventions to understand the effective causal pathways and determine which combination of behaviours should be targeted to most effectively reduce children’s risk of obesity. It is impossible to tell from an individual trial which one or combination of the many components contribute to its effectiveness, and which ones less so. Interventions in early life are indirect. They target the parent/caregiver with the intent to change their behaviour (e.g. limit screen time), which in turn aims to change child behaviour (e.g. watch less screens). This behaviour change then needs to translate to differences in a mediator variable (e.g. reduced sedentary behaviours) and in the outcome variable (e.g. healthier weight) for the intervention to be successful in preventing obesity. There is great complexity in selecting which obesity-related behaviours to target (18). For example, interventions targeting the domain diet may address parental behaviours such as limiting sugary drinks, offering vegetables, moderating portion sizes, or many others. Systematic reviews suggest that a combination of behavioural domains (e.g. diet and activity) will be more successful than addressing only one domain (e.g. diet) (5). Further, behaviours do not occur in a vacuum, but interact with and may inhibit or facilitate each other (19). Understanding relevant behaviours to target is compounded by the limited understanding of previous interventions. Often, interventions report limited detail or reporting quality is low, which leaves intervention components, pathways and target behaviours frequently underspecified (17, 20).

### Challenge 2: Heterogeneity and Complexity Impede Evidence Synthesis

Traditionally, systematic reviews and meta-analyses are regarded as the top of the evidence pyramid and are widely applied to inform healthcare policy and practice. Yet, the complexity and heterogeneity of interventions, measured outcomes and timepoints limits meaningful comparisons and impedes evidence synthesis. This makes average effect estimates of very diverse interventions difficult to interpret (21). For instance, if a trial reports obesity at age 3, another rapid weight gain at age 1, and a third BMI z-score at age 2, these results cannot be synthesised in a meta-analysis. This is particularly problematic since many clinical trials individually do not have the statistical power to show effectiveness for the ultimate clinical outcome of a healthier weight, which would require ~2000 participants (22). Conducting a trial of this size is infeasible for most researchers due to high costs of these complex interventions. This makes the combination of trials in evidence synthesis crucial. These limitations are reflected in previous reviews in this area, in which interventions were diverse and multi-faceted and their effectiveness was moderate, heterogeneous, and likely dependant on unknown intervention content (13, 14).

### Challenge 3: Population-Specificity in Changing Behaviours

Obesity in childhood affects all sections of society, but it disproportionately affects disadvantaged and minority populations, such as those experiencing lower socioeconomic position, ethnic minorities, immigrant populations, and Indigenous populations (11, 23). Many interventions and most reviews and meta-analyses of interventions currently follow a one-size-fits all approach. Yet, it is likely that different target behaviours and techniques are more relevant for different population groups. For instance, it may be of limited use to encourage certain food options to families that rely on food banks and thus have limited choice. In addition, complex instructions in a non-native language may be experienced as challenging. Elements of interventions may be experienced as culturally insensitive, for example if intervention advice differs from that of their elders (24).

### Challenge 4: Outside Influences and Fade-Out

Beneficial effects of interventions, particularly those starting in early childhood, commonly diminish over time after an intervention ends (25). This phenomenon, referred to as the fade-out effect, was apparent in the EPOCH Collaboration’s follow-up analyses (26), which found that positive intervention effects on BMI at age 2 years had dissipated by 3.5 years of age. While prolonged benefits were detected for some behaviours at 3.5 years (feeding practices, television viewing), these had diminished by age 5 in the absence of continued intervention. One reason for this may be that children undergo rapid developmental changes in the first few years of life. Therefore, interventions tailored to this life stage may not be generalisable to the later years if they are not reinforced by booster interventions specific to key developmental stages (25). Secondly, as children grow older, they are increasingly exposed to obesogenic socio-environmental influences beyond their household and family which threaten sustainability of behaviour change (27).

### Challenge 5: Scalability of Interventions

Implementation science and understanding how to integrate childhood obesity prevention into existing services have been identified as international research priorities previously (28). Even when interventions have a strong theoretical and evidence base, they frequently lack feasibility at scale (28–31). Some interventions found to be effective in a clinical trial may be too complex, resource-intensive, or situation-specific to scale up cost-effectively, and therefore can take many years to be translated into practice, if they are translated at all (29). This substantial research waste is avoidable by taking some key steps from the outset (32). For instance, building partnerships with key stakeholders, considering intervention cost and feasibility, and seeking to embed interventions within existing services (17, 33). Examples of successful at scale integration of early obesity prevention interventions include Communicating Healthy Beginnings Advice by Telephone (34) and InFANT (33). Extensive guidance arising from these examples is available elsewhere (35, 36).

## Proposed Solutions

**Figure 2:**
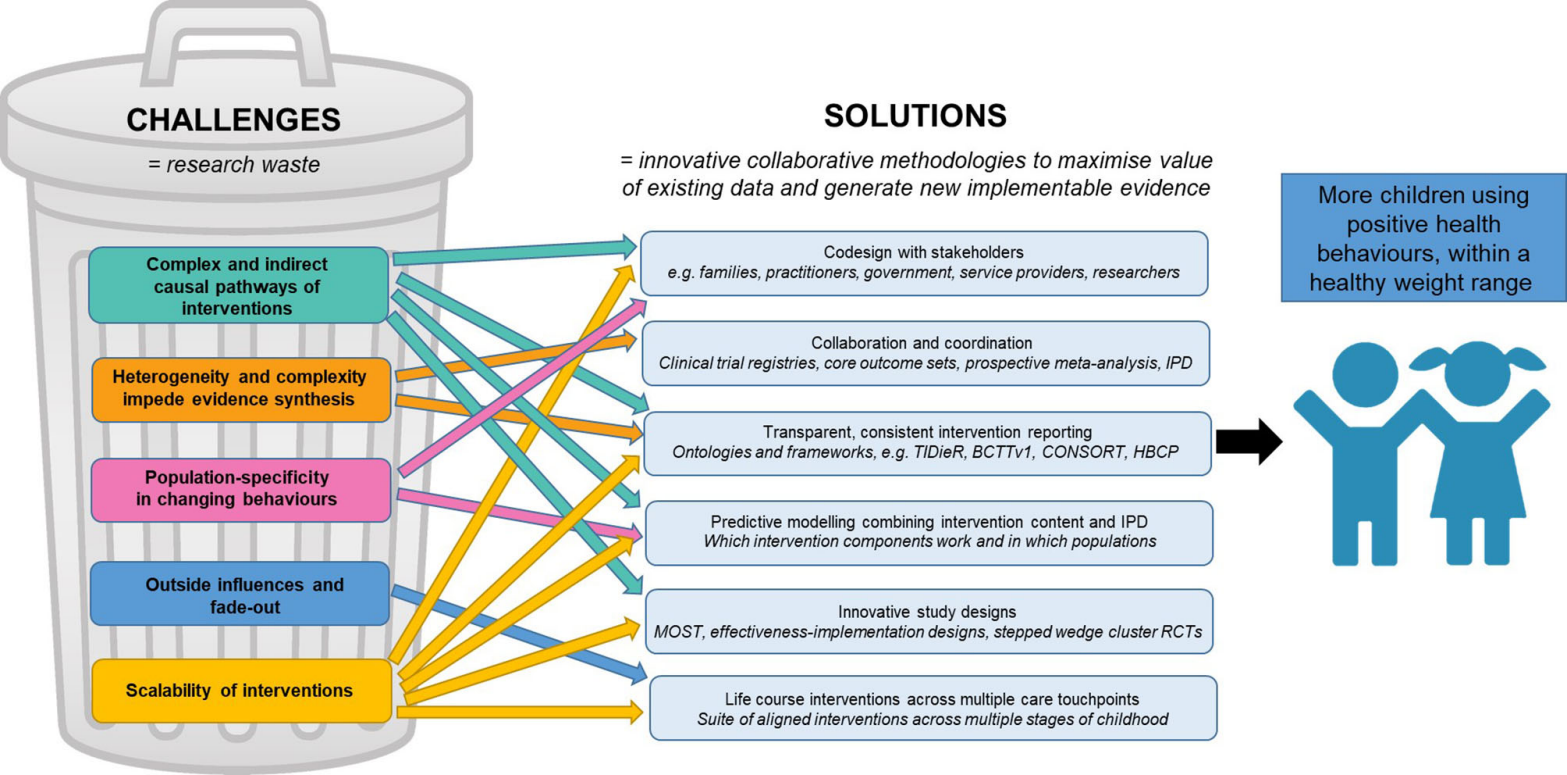
Proposed solutions to challenges in early childhood obesity prevention interventions. Associations and interactions are hypothesised to be present between each of the challenges, as well as between each of the solutions. BCTTv1, Behaviour Change Technique Taxonomy version 1; CONSORT, Consolidated Standards of Reporting Trials; HBCP, Human Behaviour Change Project; IPD, individual participant data; MOST, Multiphase Optimisation Strategy; RCTs, randomised controlled trials; TIDieR, Template for Intervention Description and Replication.

**Figure 3:**
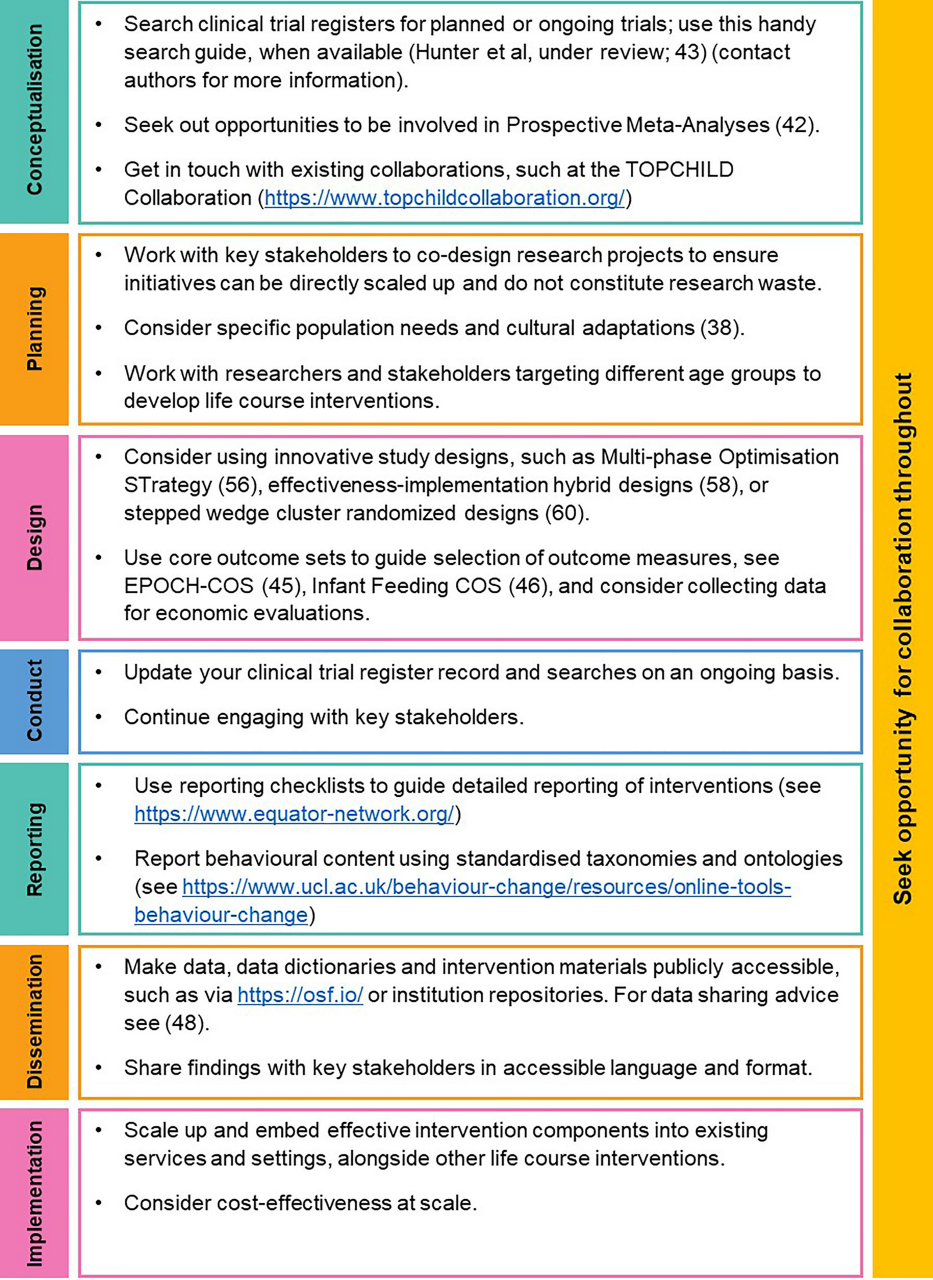
How to apply proposed solutions throughout the research process.

We present several proposed solutions to collectively address these challenges in early childhood obesity prevention (**Figure 2**). Guidance to apply the proposed solutions throughout the research process is summarised in **Figure 3** and seeks to serve as a quick reference guide for researchers.

### Solution 1: Codesign With Stakeholders

Stakeholder consultation and consideration of historical, political and structural context are crucial from the outset to develop effective interventions for adoption at scale (17, 37, 38). Codesign, also termed co-production or consumer engagement, has been widely popularised in health research as a potential solution to bridge the gap between research and practice and reduce waste arising from interventions that are never implemented (32). Codesign involves meaningfully working with key stakeholders to understand the world through their lens (37, 39). Key stakeholders in childhood obesity prevention include families as beneficiaries, and end users of practitioners (e.g. health care workers, educators) and policy makers. Engagement with stakeholders can ensure interventions are culturally sensitive and appropriate (24, 40). Considerations of health literacy, language barriers, access to and trust in health services, are also important to reach and engage marginalised populations (7). For instance, the Healthier Together community prevention program used an iterative, participatory and experience-based process with consumers, cultural advisors and health professionals, to design a culturally appropriate intervention promoting acceptability, effectiveness, and equity (41).

### Solution 2: Collaboration and Coordination

There are several approaches and methodologies that can facilitate collaboration and coordination between researchers. From early planning and throughout all stages of the intervention process, researchers should conduct regular searches of clinical trial registries. Searching registries allows researchers to monitor emerging evidence and opportunities for collaboration. For instance, investigators may identify similar planned or ongoing studies and coordinate their efforts in a prospective meta-analysis (42). Searching registers may also inform research prioritisation by highlighting areas where more research may (or may not) be needed and enable access to data for inclusion in reviews. Comprehensive guidance on how to harness the benefits of registers has been developed (Hunter et al, under review) and is currently available upon request in summary form (43).

Another collaborative method is the application of core outcome sets (COS). Core outcome sets are a minimum set of recommended outcomes in studies of a certain topic area, agreed by key stakeholders (44). A core outcome set for early childhood obesity prevention (EPOCH-COS) is in development (45), and one for infant feeding already exists (46). If applied widely by individual studies, COS make evidence synthesis more powerful and less heterogeneous. Development of specific core outcome measurement tools would further reduce heterogeneity. Another useful methodology is prospective meta-analysis, whereby researchers decide to collaborate and agree on key protocol elements, *before* results of their individual studies are known (42). This allows detailed harmonisation of outcomes, i.e. researchers agree to collect the same outcomes using the same measures at the same timepoints (22). In the EPOCH prospective meta-analysis, the number of available outcomes that could be synthesised upon completion increased from 18% to 91% after deciding to collaborate in a prospective meta-analysis, drastically improving statistical power (22). A related collaborative methodology for evidence synthesis is individual participant data meta-analysis, which involves the row-by-row collation of raw data. This is regarded as the gold standard for meta-analysis, as it allows more complex analyses using the most current and comprehensive data (47). Data sharing is crucial for individual participant data meta-analyses to be conducted (48). In particular, it enables participant-level subgroup analyses, allowing exploration such as if an intervention works for certain groups (e.g. priority populations), which is important for health equity.

### Solution 3: Transparent, Consistent Intervention Reporting

An improved understanding of intervention content provides a first step toward designing more effective and efficient interventions. Clear, transparent reporting of intervention development, content and implementation assists in understanding how interventions do, or do not, work to change behaviour (20). There have been several advances in the creation or adaption of reporting checklists (49, 50), taxonomies (51) and ontologies (52). Adoption of such tools will increase transparency of intervention behavioural content and assist evidence synthesis. Of emerging importance is providing access to, or detailed reporting of, unpublished intervention materials, for instance *via* supplementary files or open science repositories. Access to such materials (e.g. facilitator manuals, parent resources) enabled identification of substantially more behaviour change techniques than from published materials when relevant taxonomies were retrospectively applied (17, 53). While greater detail in reporting of intervention components is progress towards a solution, innovative methods are then required, such as predictive modelling, to disentangle effective intervention components (Solution 4).

### Solution 4: Predictive Modelling Combining Intervention Content and Individual Participant Data

The question of which components drive behaviour change can be addressed by combining individual participant data with intervention components in a predictive modelling approach. These models can quantitatively explore which intervention components are associated with effectiveness, compared with usual care, particularly for priority population groups. This methodology is being applied in the TOPCHILD Collaboration^1^, where trial investigators around the world are working together to find the best interventions for different population groups (16, 54). Importantly, these quantitative methods should be combined with qualitative information obtained from close collaboration with key stakeholders on the types of components that are feasible, scalable and cost-effective. This information can be used to co-produce recommendations, highlighting the critical or active ingredients of interventions. Findings can guide translation of the most promising interventions into routine practice, as well as the development of future optimum obesity prevention interventions.

### Solution 5: Innovative Study Designs

There are a range of study designs that can address resource-intensiveness of randomised controlled trials (55), accelerate testing of new interventions and their components, and bridge the intervention-to-practice gap. Firstly, the Multiphase Optimization STrategy (MOST) (56) allows several intervention packages to be tested through one RCT, combining an optimisation phase, to find an optimised intervention package, with an evaluation phase, to test this package (56). This approach was used to evaluate nine intervention packages using 16 different experimental conditions simultaneously within a 5-week period to quickly test and refine content for a responsive parenting intervention to prevent obesity (57). Secondly, effectiveness-implementation hybrid designs seek to improve the translation of interventions by evaluating implementation strategies at the same time as assessing the trial effect (58). The MINISTOP 2.0 mobile app study tested intervention effectiveness concurrently with the potential scale up through routine child health care (59). Finally, stepped wedge cluster randomised trial designs allow all groups to receive an intervention, by first acting as control groups, randomising the timing of intervention initiation. This allows for greater reach of an intervention (60). For example, the Communities for Healthy Living trial is testing the roll out of an integrated service with three times the sites over a three-year period, compared to the parallel group pilot (61). Use of these different study designs can accelerate intervention testing and evaluate interventions in ‘real world’ contexts to reduce challenges of implementation at scale.

### Solution 6: Life Course Interventions Across Multiple Care Touchpoints

There is a need for strategies to address fade-out of early intervention benefits (25), to keep children on a healthy growth trajectory and sustain greater return on investment. This may take the form of post-intervention maintenance strategies, which have shown promise following childhood obesity treatment interventions (62). Though post-intervention maintenance strategies are under-researched in the prevention area. Additionally, a life course approach may reduce fade-out, whereby a suite of complementary interventions are implemented across multiple stages of childhood and across multiple health and community care touchpoints that support children and their care providers (63). This will support persistent positive behaviours and sustained beneficial effects (64–66). These interventions should be combined with the widely advocated systems approach to address sociocultural, political, economic and environmental influences contributing to obesity risk (67, 68).

## Discussion

In this perspective, we describe key challenges of early childhood obesity prevention, and a suite of solutions throughout the research process (**Figure 3**). While the presented solutions are diverse, they share the underlying theme of requiring collaboration and coordination. Researchers need to work together to harmonise their studies and share data and intervention content. Researchers need to collaborate across disciplines (e.g. statistics, public health) and expertise (e.g. qualitative, advanced quantitative). For interventions to be acceptable, feasible and scalable they need to be codesigned with stakeholders, and integrated with other policies and programs, to ensure their effectiveness is sustained across the life course.

Achieving the required level of collaboration and coordination is difficult. The traditional academic ecosystem requires researchers to compete for scarce funding and positions. Cross-discipline communication is complicated, and resources and skills are required to coordinate big and diverse project teams. Fortunately, there are a range of positive examples showing successful collaboration is possible, including the EPOCH Collaboration, the TOPCHILD Collaboration, and the HeLTI Consortium (15, 16, 54, 69). Importantly, all of these large collaborations were awarded competitive grants to support their resource-intensive coordination, underlining the importance of public funding schemes to support coordinated approaches. Another positive development is emerging structural initiatives to improve collaboration in the research ecosystem. One example is the proposal for contributorship (70), and the concept of data authorship, giving credit to researchers every time their data are re-used (71). In addition, funders and universities have started moving away from solely looking at numbers of publications, and toward impact of research work to judge academic performance. These criteria may encourage open science *via* sharing of data and knowledge and reward the impact of successful codesign and translation into practice.

The COVID-19 pandemic has shown us the importance of global collaboration. There have been many calls and successful examples of rapid coordination, collaboration and translation into practice (72, 73). It is imperative to apply these learnings into other research fields, including childhood obesity prevention.

In conclusion, childhood obesity prevention is complex, and can only be addressed by extensive collaboration and coordination across sectors, disciplines, policy and practice end users. Researchers in this area have made enormous progress in addressing challenges. If the presented research solutions are combined and applied, together with policy, practitioner and consumer perspectives as a collaborative global community, the quest to set children on a lifelong healthy behaviour trajectory can be realised.

## Data Availability Statement

The original contributions presented in the study are included in the article. Further inquiries can be directed to the corresponding author.

## Author Contributions

AS, BJ, RG, and KH conceived the concepts presented in this perspective. AS, BJ, and KH conceived the manuscript structure and drafted the manuscript. RG critically reviewed the manuscript. All authors contributed to the article and approved the submitted version.

## Funding

BJ is supported by funding from the NHMRC Ideas Grant TOPCHILD (Transforming Obesity Prevention for CHILDren): Looking into the black box of interventions (GNT1186363). This work was supported by the Early Prevention of Obesity in Childhood, NHMRC Centre for Research Excellence (GNT1101675).

## Conflict of Interest

AS, BJ, RG, and KH are part of the EPOCH and EPOCH Translate Centres for Research Excellence. AS is the chair of the TOPCHILD Collaboration. BJ and KH are co-deputy chairs of the TOPCHILD Collaboration. AS and KH are affiliated with the Australian and New Zealand Clinical Trials Register. AS is the co-convener and primary contact of the Cochrane Prospective Meta-Analysis Methods Group. KH is the associate convener of the Cochrane Prospective Meta-Analysis Methods Group.

## Publisher’s Note

All claims expressed in this article are solely those of the authors and do not necessarily represent those of their affiliated organizations, or those of the publisher, the editors and the reviewers. Any product that may be evaluated in this article, or claim that may be made by its manufacturer, is not guaranteed or endorsed by the publisher.

## Footnotes

1
www.topchildcollaboration.org
